# Study on the Mechanism of the Blood-Glucose-Lowering Effect of Collagen Peptides from Sturgeon By-Products

**DOI:** 10.3390/md19100584

**Published:** 2021-10-19

**Authors:** Yukiho Sasaoka, Taichi Takagi, Shunta Michiba, Yohei Yamamoto, Yuya Kumagai, Hideki Kishimura

**Affiliations:** 1 Marine Chemical Resource Development, Graduate School of Fisheries Sciences, Hokkaido University, Hakodate, Hokkaido 041-8611, Japan; tkg.taichi@gmail.com (T.T.); shun_ta_soft@yahoo.co.jp (S.M.); bknb626@gmail.com (Y.Y.); 2 Laboratory of Marine Chemical Resource Development, Faculty of Fisheries Sciences, Hokkaido University, Hakodate, Hokkaido 041-8611, Japan; yuyakumagai@fish.hokudai.ac.jp

**Keywords:** sturgeon by-products, collagen peptides, blood glucose level, suppression of absorption, DPP-IV, GLP-1, insulin

## Abstract

In a previous study, we found that the collagen peptides prepared from the by-products of Bester sturgeon had an inhibitory effect on elevated blood glucose levels in a glucose tolerance test with ICR mice. In the present study, we examine the mechanism of the effect of sturgeon collagen peptides (SCPs) in detail. When glucose was orally administered to mice along with the SCPs, it was found that the glucose remained in the stomach for a longer time. In the above tests, the amount of glucose excreted in the feces of mice also increased. On the contrary, it was revealed that the SCPs have a dipeptidyl-peptidase-IV (DPP-IV) inhibitory ability in an in vitro test. In subsequent oral and intravenous glucose administration tests, glucagon-like peptide-1 (GLP-1) and insulin levels in the blood of mice were maintained at high levels. These results suggested the following three mechanisms: SCPs slow the rate of transportation of glucose from the stomach into the small intestine, resulting in delayed glucose absorption; SCPs suppress the absorption of glucose in the small intestine and excrete it from the body; SCPs inhibit DPP-IV in the blood and maintain a high GLP-1 level in blood, which in turn stimulates insulin secretion.

## 1. Introduction

The number of diabetics in the world is increasing due to cultural and social lifestyle changes associated with economic development. According to a report by the International Diabetes Federation, the total number of diabetic patients in the world was 463 million in 2019, and will rise to 578 million by 2030, and 700 million by 2045 [1]. In addition, it is expected that the number of diabetic patients will reach 628.6 million by 2045, making the prevention of diabetes an urgent issue [1]. Over 90% of diabetic patients have type 2 diabetes mellitus, which is closely related to insulin, a glucose metabolism hormone secreted by the pancreas. Insulin is the only hormone that lowers blood glucose levels by causing blood glucose to be taken up by muscle and fat cells. However, genetic factors such as race and ancestry, environmental factors such as overeating, stress, obesity, smoking, and a lack of exercise, and aging factors induce a decrease in insulin secretion from the pancreas and a decrease in the efficiency of glucose uptake by muscles (insulin resistance) [2,3]. In addition, loss of muscle mass due to aging and lack of exercise leads to a decrease in glucose uptake, causing a constant state of hyperglycemia [1,4]. Hyperglycemia damages the heart, blood vessels, eyes, kidneys, and nerves, which may lead to blindness, limb amputation, kidney failure, and death [1,5]. As hypoglycemic agents, glinides and sulfonylureas are commonly used. However, these drugs are used to treat diabetes, not to prevent the onset of diabetes.

In recent years, to prevent type 2 diabetes, it has been recommended to reduce the intake of sugar, carbohydrates, and fat, and to improve the content of the diet by incorporating vegetables and fruits [4]. For example, the consumption of fish and seaweed has been shown to be associated with a reduced risk of developing type 2 diabetes [6,7,8]. These food components prevent a progression to type 2 diabetes by stopping a rapid increase in postprandial blood glucose levels and reducing the burden on the pancreas. If postprandial blood glucose levels can be easily controlled with food components, it may be the most effective means of preventing diabetes. In a previous study, we isolated an acid- and heat-stable trypsin inhibitory peptide from the viscera of Japanese common squid (*Todarodes* *pacificus*) [9]. We reported that the squid trypsin inhibitor did not inhibit elevated blood glucose in healthy Wistar rats, but only in GK rats that were a model of type 2 diabetes, and its effect was related to an improvement in insulin secretion in GK rats [9,10]. It was concluded that the enhanced insulin secretion was due to the fact that the inhibitor reached the small intestine without being digested by proteolytic enzymes and increased the secretion of digestive hormones (gastrin, gastrokine, cholecystokinin) [11].

Sturgeons inhabit a wide range of aquatic areas, including large rivers, such as the Amur (Heilongjiang), Yangtze, and Danube, and lakes and inland seas of the Northern Hemisphere [12,13]. It is one of the largest freshwater fish species, growing up to 8 m in length and more than 1 t in weight, depending on the species [12,13]. They are large-sized and have many edible parts, therefore, they are an important source of protein, especially along the Caspian Sea coast and in Russia [14]. In addition, swim bladders and salted egg products have high economic value as isinglass and caviar, respectively [14,15]. In Japan, sturgeon have been farmed since the 1980s for the purpose of harvesting eggs and meat, while other parts, such as skin, fin, and bone, are by-products that are not fully utilized [16]. However, these parts are rich in collagen [17]. We previously prepared collagen peptides from Bester sturgeon (*Huso* × *Acipenser ruthenus*) by-products (the skin, fin, and bone), and investigated the inhibitory effect of the sturgeon collagen peptides (SCPs) on elevated blood glucose levels [18]. As a result, we found that the SCPs showed an inhibitory effect on elevated blood glucose levels in a glucose tolerance test with ICR mice. We clarified that most of the peptides in the SCPs, fractionated by gel filtration and reversed-phase HPLC, consisted of Gly-X-Y (X and Y are optional amino acid residues) repetitive sequences, which are common to the triple helical region of the collagen molecules [18]. Therefore, in this study, we further investigated the mechanism of the effect of the SCPs in detail.

## 2. Results and Discussion

### 2.1. Effect of the SCPs on α-Glucosidase and Invertase Abilities

Inhibition of α-glucosidase in the small intestine by food-derived components is widely known as one of the mechanisms for the suppression of blood glucose elevation.

**Figure 1:**
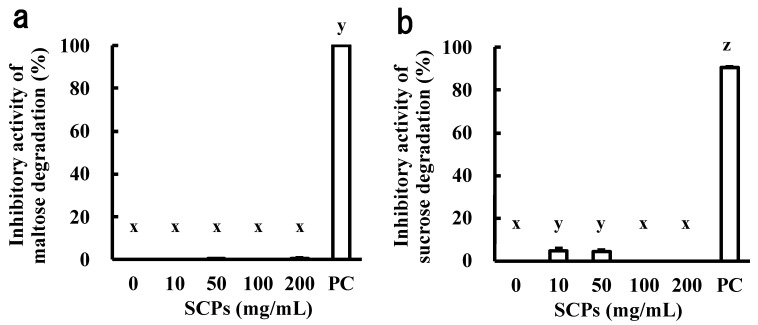
Effect of the SCPs on α-glucosidase and invertase abilities. (**a**) Inhibitory activity of the SCPs on α-glucosidase; (**b**) inhibitory activity of the SCPs on invertase. Bars represent standard errors. There is a significant difference between different signs (x, y, and z) (*p* < 0.05; Tukey–Kramer test). PC: 1-deoxynojirimycin. Data are expressed as mean ± SEM (*n* = 3–4).

In this study, we investigated the inhibitory activity of the SCPs on α-glucosidase and invertase from rat small intestine. However, the SCPs did not inhibit either enzyme (Figure 1a,b).

### 2.2. Effect of the Orally Administrated SCPs on Residual Glucose Content in the Stomach in the Oral Glucose Tolerance Test (OGTT)

**Figure 2:**
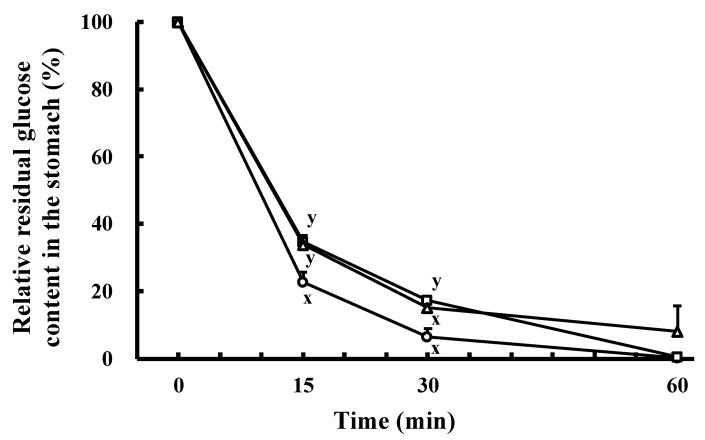
Effect of the SCPs on glucose retention in the stomach in the OGTT in normal mice. Relative residual glucose content in the stomach. Open circles: the control group; open squares: the SCP group; open triangles: the porcine collagen peptides group. Data are expressed as mean ± SEM (*n* = 2 at 0 min, *n* = 3 at 15 min, *n* = 4 at 30 min, *n* = 2 at 60 min). There is a significant difference between different signs (x and y) (*p* < 0.05; Tukey–Kramer test).

We hypothesized that prolonged glucose residence time in the stomach would reduce the rate of glucose influx from the stomach to the small intestine and suppress the rapid increase in blood glucose. Therefore, we investigated the effect of the orally administrated SCPs on the residence time of glucose in the stomach. As shown in Figure 2, the glucose existence rates at 15 and 30 min after oral administration in the SCP group (34.6 ± 1.9 and 17.3 ± 0.8%, respectively) were significantly higher than those of the control group (22.7 ± 3.1 and 6.6 ± 2.4%, respectively). To further confirm this result, we carried out an experiment using porcine collagen peptides. The results showed that the amount of glucose remaining in the porcine collagen peptides group was also higher than that of the control group, and the glucose existence rates at 15 min (33.8 ± 1.5%) were significantly higher than those of the control group (Figure 2). In general, the transfer rate from the stomach to the small intestine slows down with the amounts of solids in the sample [19,20]. The soluble solids in the SCP group sample (23.3%) were higher than those of the control group sample (13.3%). In addition to the physical effect, it was reported that when proteins are ingested, the transport of proteins from the stomach to the small intestine is inhibited to allow for adequate protein digestion in the stomach [21]. In other words, it has been reported that collagen peptides upregulate GLP-1 secretion, which delays gastric emptying [22,23]. Therefore, it is possible that the SCPs may also delay gastric emptying indirectly via GLP-1 secretion. For these two reasons, it was thought that the glucose in the SCP group sample remained in the stomach along with the SCPs for a longer time.

### 2.3. Effect of the Orally Administrated SCPs on Glucose Absorption in the Small Intestine in the OGTT

**Figure 3:**
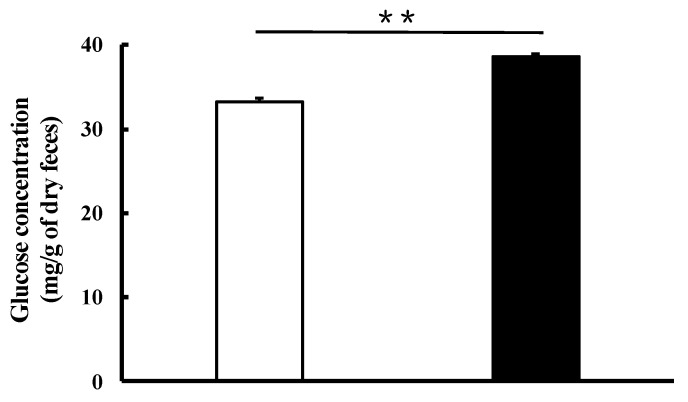
Effect of SCPs on glucose absorption in the small intestine in OGTT in normal mice. The white column: the control group; the black column: the SCP group. Data are expressed as mean ± SEM (*n* = 8). Values with asterisk indicate statistically significant difference (** *p* < 0.01; Student’s *t*-test).

To investigate the effect of SCPs on glucose absorption in the small intestine, the glucose excretion rate in feces was measured. After passing through the small intestine, carbohydrates are metabolized by intestinal microorganisms in the colon and cecum. In our earlier studies, we examined the amount of glucose excreted in feces using rats, but we were unable to detect glucose in feces (data not shown). Hence, in the present study, we used ICR mice, which have a smaller cecum than rats. The vertical axis in Figure 3 shows the glucose excretion rate in feces when the glucose dose weight (2.0 g/kg body weight) is set to 100%. As a result, 33.2 mg/g of dry feces (6.4 ± 0.1% of the glucose dose) was detected in the feces in the distilled water group, and 38.6 mg/g of dry feces (9.3 ± 0.1% of the glucose dose) was detected in the SCP group. The glucose excretion rate of the SCP group was significantly (*p* < 0.01) higher than that of the distilled water group. These results suggested that the orally administered SCPs inhibited glucose absorption in the small intestine, resulting in increased glucose excretion.

### 2.4. Effect of the Orally Administrated SCPs on Blood Glucose and Insulin Levels in the Intraperitoneal Glucose Tolerance Test (IPGTT)

**Figure 4:**
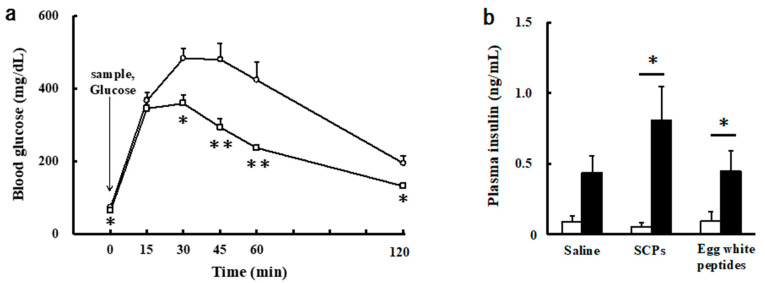
Effect of the orally administrated SCPs on blood glucose and insulin levels in the IPGTT in normal mice. (**a**) Blood glucose levels when the SCPs and glucose were administered orally and intraperitoneally, respectively. Open circles: the control group; open squares: the SCP group. Data are expressed as mean ± SEM (*n* = 7). Values at the same time-point with asterisk indicate statistically significant difference (* *p* < 0.05, ** *p* < 0.01; Mann–Whitney *U* test). (**b**) Plasma insulin levels when sample (saline, SCPs, egg white peptides) and glucose were administered orally and intraperitoneally, respectively. The white columns: the values at 0 min; the black columns: the values at 15 min. Data are expressed as mean ± SEM (*n* = 5–6). Values with asterisk indicate statistically significant difference (*p* < 0.05; Wilcoxon rank sum test).

As mentioned above, it was found that the SCPs suppress the rise in blood glucose level by delaying and inhibiting the absorption in the small intestine in the OGTT. Thus, we measured blood glucose levels when the SCPs and glucose were administered orally and intraperitoneally, respectively. As shown in Figure 4a, in the IPGTT, the blood glucose levels in the SCP group were significantly lower than those of the control groups at 30, 45, 60, and 120 min. Although, there was no significant difference between the control and the SCP groups in terms of blood glucose levels at 15 min in the IPGTT, the blood glucose levels of the SCP group were lower than those of the control group after 15 min. The result, that the blood glucose levels of both groups rose similarly between 0 and 15 min in the IPGTT, indicated that the SCPs may have suppressed the rise in blood glucose level by delaying glucose transportation in the stomach and inhibiting glucose absorption in the small intestine in the OGTT. From the results, we speculated that the SCPs not only delay and inhibit absorption in the small intestine, but also may suppress the increase in blood glucose levels in vivo.

Moreover, we examined the effect of the orally administrated SCPs on blood insulin level in the IPGTT. The insulin level was 4.9 times higher than that of the basal level in the control group, whereas the level was 15.4 times higher than that of the basal level in the SCP group (Figure 4b). Additionally, the insulin level of the SCP group at 15 min was 1.8 times higher than that of the control group. On the other hand, the insulin level was 4.8 times higher than that of the basal level in the egg white peptide group, and the insulin level of the egg white peptide group at 15 min (0.449 mg/dL) was almost the same as that of the control group (0.439 mg/dL). The results supported the above hypothesis that SCPs suppress the increase in blood glucose levels by insulin secretion, in addition to delaying glucose transportation in the stomach and inhibiting glucose absorption in the small intestine. Furthermore, its insulin secretory promoting effect was more effective than that of egg white peptides.

### 2.5. Effect of the Intravenously Injected SCPs on Blood Glucose and Insulin Levels in the IPGTT

As described before, it was found that the SCPs have the potential to suppress the increase in blood glucose levels through the secretion of insulin, in addition to delaying glucose transportation in the stomach and inhibiting glucose absorption in the small intestine. In general, gelatin and collagen peptides have resistance to enzymatic degradation [24,25]. We further investigated the effect of the intravenously injected SCPs on blood glucose and insulin levels in the IPGTT.

First, before the intravenous injection, we measured the amounts of coexistent endotoxin in the SCP samples that were prepared on different days. As shown in Table 1, both samples had less than 0.1 ng/mg (SCPs-1: 0.014 ng/mg; SCPs-2: 0.100 ng/mg), suggesting that there was little effect of the endotoxin.

**Figure 5:**
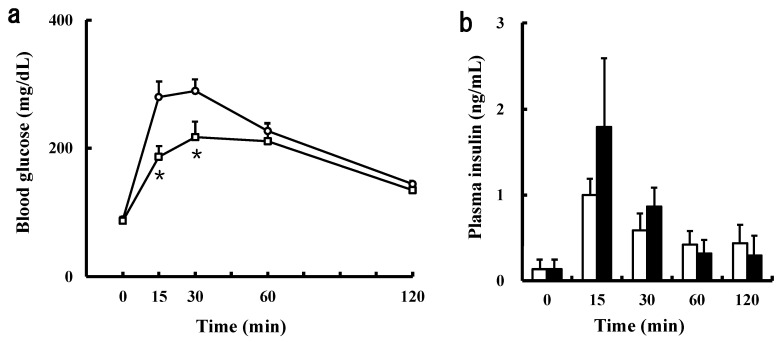
Effect of the intravenously injected SCPs on blood glucose and insulin levels in the IPGTT in normal mice. (**a**) Blood glucose levels when the SCPs and glucose were injected intravenously and intraperitoneally, respectively. Open circles: the control group; open squares: the SCP group. Data are expressed as mean ± SEM (*n* = 6–8). Values at the same time-point with asterisk indicate statistically significant difference (* *p* < 0.05; Mann–Whitney *U* test). (**b**) Plasma insulin levels when the SCPs and glucose were injected intravenously and intraperitoneally, respectively. The white columns: the control group; the black columns: the SCP group. Data are expressed as mean ± SEM (*n* = 3–5).

Next, we examined the effect of the intravenously injected SCPs on blood glucose and insulin levels in the IPGTT. As indicated in Figure 5a, the blood glucose levels in the SCP group were lower than those of the control group, and the levels at 15 and 30 min were significantly lower than those of the control group. Furthermore, the insulin levels in the SCP group at 15 and 30 min were higher than those of the control group (Figure 5b). These results suggested that SCPs, or their degradation products, stimulate the secretion of insulin after it is absorbed from the small intestine and transferred to the veins. Concerning absorption of collagen peptides, some reports have indicated that hydroxyproline (Hyp)-containing collagen peptides increase in human plasma and urine after collagen intake [26,27].

### 2.6. Effect of the SCPs on DPP-IV Activity

**Figure 6:**
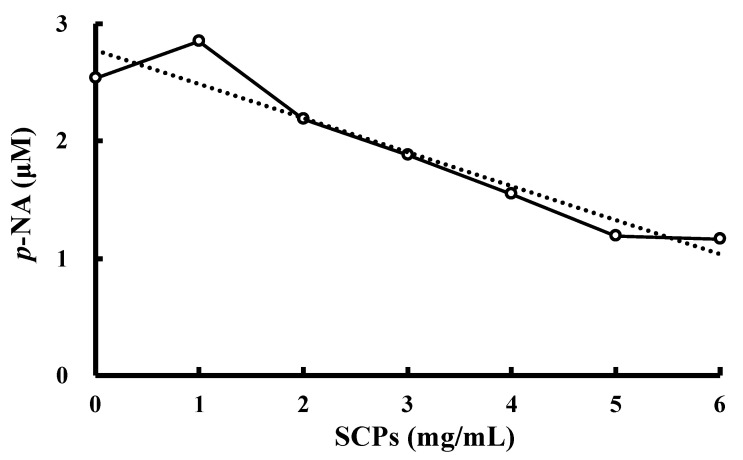
The relationship between the concentrations of the SCPs and the degradation product (*p*-NA). Error bars are not shown because the SEM is smaller than the height occupied by the symbol representing the mean value (*n* = 3). The dotted line indicates the regression line: y = −0.2904x + 2.78 (*R*^2^ = 0.9109).

Glucagon-like peptide-1 (GLP-1) is synthesized in the L-cells of the small intestine as a glucagon-containing precursor peptide, and is involved in the inhibition of glucagon secretion and the promotion of insulin secretion in the pancreas [28]. The N-terminal dipeptides of the GLP-1 are cleaved by DPP-IV (EC 3.4.14.5), and the cleaved GLP-1 acts as an antagonist to the above physiological effects [29]. Therefore, the inhibition of DPP-IV is an effective way to prevent and treat diabetes. DPP-IV is an enzyme that specifically recognizes peptides with a Pro residue (or, to a lesser extent, an Ala residue) at the second position from the N terminus, and cleaves the C-terminal side of the Pro (or Ala) to release the dipeptide. Li-Chan et al. also reported that a Pro residue was determined as the second N-terminal residue, and was flanked by Gly and Ala in the DPP-IV inhibitory peptides from the skin collagen of Atlantic salmon [30]. In a previous study, we identified several peptides with Pro or Ala residues at the second position from the N terminus in the SCPs, such as Ala-Ala-Gly-Pro-Hyp-Gly, Gly-Pro-Gly-Gly-Pro-Ala, Gly-Pro-Leu-Gly-Pro-Ala, Ala-Pro-Ala-Gly, and Ala-Pro-Asn-Pro-Phe-Arg-His-Lys [18]. Hence, we predicted that the SCPs would have DPP-IV inhibitory activity. Then, in this study, we investigated the DPP-IV inhibitory activity of the SCPs. As a result, we found the SCPs inhibited the formation of the degradation product of *p*-nitroaniline *(p*-NA) from the substrate (H-Gly-Pro-*p*-NA) by DPP-IV in a concentration-dependent manner, and its IC_50_ value was 934 μg/mU (Figure 6, Table 2). Collagen peptides in the previous reports were prepared with enzymes other than papain (Table 2). For example, Flavourzymes and Esperases are mixtures of several enzymes produced by microorganisms, and the peptides prepared by the enzymes are presumed to be composed of different amino acid sequences because of their different cleavage specificities. However, the SCPs showed almost the same inhibitory effect as those of other fish collagen peptides (Table 2). On the other hand, the IC_50_ of SCPs was higher than those of synthetic peptides (Gly-Pro-Ala-Glu and Gly-Pro-Gly-Ala). SCPs are a mixture of peptides of various sequences and molecular weights. As a result, it was presumed that a large number of peptides with low DPP-IV inhibitory activity were also contained in the SCPs. The average molecular weight of the SCPs was approximately 1400, and many Gly-Pro and Gly-Ala sequences were detected in the internal sequence of the SCPs [18]. Therefore, further hydrolysis of the SCPs would increase the content of peptides with Gly-Pro and Gly-Ala sequences at the N terminus, which would in turn enhance its DPP-IV inhibitory activity.

### 2.7. Effect of the Intravenously Injected SCPs on Blood Glucose and GLP-1 Levels in the IPGTT

**Figure 7:**
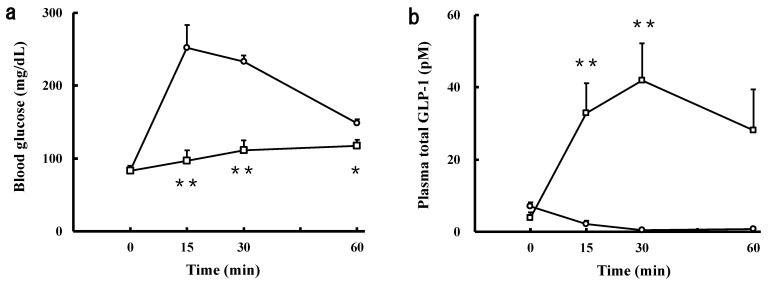
Effect of the intravenously injected SCPs on blood glucose and GLP-1 levels in the IPGTT in normal rats. (**a**) Blood glucose levels when the SCPs and glucose were injected intravenously and intraperitoneally, respectively. Open circles: the control group; open squares: the SCP group. Data are expressed as mean ± SEM (*n* = 6). Values at the same time-point with asterisk indicate a statistically significant difference (* *p* < 0.05; ** *p* < 0.01; Student’s *t*-test). (**b**) Plasma GLP-1 levels when the SCPs and glucose were injected intravenously and intraperitoneally, respectively. Open circles: the control group; open squares: the SCP group. Data are expressed as mean ± SEM (*n* = 6). Values at the same time-point with asterisk indicate a statistically significant difference (** *p* < 0.01; Mann–Whitney *U* test).

As described above, it was found that the SCPs are capable of inhibiting DPP-IV. Since the SCPs delay gastric emptying, as indicated in the previous paragraph, we speculated that the effect of GLP-1 might be superior to that of GIP [32]. Hence, we investigated the effect of the intravenously injected SCPs on blood GLP-1 levels in the IPGTT. As shown in Figure 7a, the blood glucose levels in the SCP group were lower than those of the control group, and the levels at 15, 30, and 60 min were significantly lower than those of the control group. Moreover, the GLP-1 levels in the SCP group at 15 and 30 min were maintained higher than those in the control group (Figure 7b). The L-cells, which are located between the duodenum and ileum, are directly and indirectly sensed by food-derived ingredients and their digested products, followed by secreting the GLP-1 [32,33,34]. Therefore, it was suggested that the SCPs stimulate the secretion of GLP-1 from L-cells, followed by the GLP-1 stimulating the secretion of insulin, resulting in a decrease in blood glucose level. Iba et al. reported that CP increases the level of active GLP-1 [22]. We also found that SCPs increased blood insulin levels, and that SCPs showed DPP-IV inhibitory activity in vitro. Based on these facts, we speculated that SCPs enhance the secretion of GLP-1, which in turn increases the level of active GLP-1, which is not degraded by DPP-IV.

**Figure 8:**
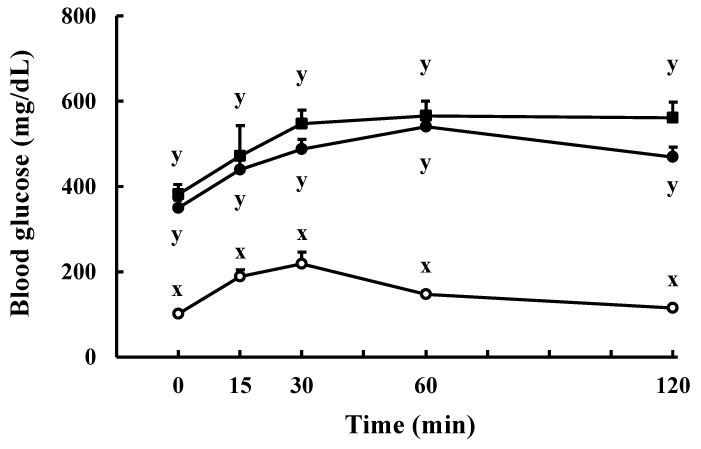
Effect of the intravenously injected SCPs on blood glucose levels of STZ rats in the IPGTT. Open circles: the control group using SD rats; closed circles: the control group using STZ rats; closed squares: the SCP group using STZ rats. Data are expressed as mean ± SEM (*n* = 3–4). There is a significant difference between different signs (x and y) (*p* < 0.05; Tukey–Kramer test).

To confirm the above deduction, we tested the temporal changes in blood glucose levels of type 1 diabetic model rats (STZ rats) after intravenous administration of the SCPs and intraperitoneal glucose loading. The SCPs did not lower the blood glucose levels in rats without insulin secretory capacity (Figure 8). It was also reported that the hydrolysates prepared from the skin collagens of Atlantic salmon and porcine prevent the degradation of GLP-1 by DPP-IV, thereby maintaining high blood levels of GLP-1, which in turn promote insulin secretion and improve postprandial hyperglycemia [22,35,36].

## 3. Materials and Methods

### 3.1. Materials

Heparin sodium, 1-deoxynojirimycin, aprotinin from bovine lung, streptozotocin, and Glucose CII-Test Wako were purchased from the FUJIFILM Wako Pure Chemical Corporation (Osaka, Japan). Papain (EC 3.4.22.2, 1:350) and porcine-skin-derived gelatin were purchased from Sigma-Aldrich Co. LLC (St. Louis, MO, USA) and the FUJIFILM Wako Pure Chemical Corporation (Osaka, Japan). An LBIS Mouse Insulin ELISA Kit (AKRIN-011T) was purchased from the FUJIFILM Wako Shibayagi Corporation (Gunma, Japan). A Multi Species GLP-1 Total ELISA kit (EZGLP1T-36K) was purchased from Merck Millipore (Burlington, MA, USA). Saline was purchased from Otsuka Pharmaceutical Co., Ltd. (Tokyo, Japan). Ethylenediamine-*N,N,N′,N′*-tetraacetic acid, tripotassium salt, and dihydrate (EDTA 3K) were purchased from Dojindo (Kumamoto, Japan). A DPP-IV Drug Discovery Kit (BML-AK499) was purchased from Enzo Life Sciences, Inc. (Farmingdale, NY, USA). An Endospecy^®^ ES-24S Kit was purchased from the Seikagaku Corporation (Tokyo, Japan). Six-week-old male ICR mice and SD rats were purchased from Charles River Laboratories (Yokohama, Japan). They were maintained under controlled temperature (23 ± 1 °C), humidity (50 ± 10%), and 12 h-light/dark cycle (light: 08:00–20:00), with free access to deionized water and standard chow (MF diet, Oriental Yeast Co., Tokyo, Japan). All procedures for the use and care of the mice were approved by Institutional Animal Care and Use Committee of National University Corporation Hokkaido University.

The SCPs were prepared as previously described [18]. The skin, fin and bone were harvested from Bester sturgeon and pretreated by soaking in 1% NaCl for 48 h and 0.2 M NaOH, 0.2 M HCl, and distilled water for 24 h in series. Then, the pretreated materials were hydrolyzed by 0.2 wt % of papain to the SCP at 50 °C for 5 h. After centrifugation at 4 °C, 10,000× *g* for 10 min, the supernatant was lyophilized. Then, lipids in the hydrolysates were removed by soaking in 99.5% ethanol for 3 h, and delipidated materials were dried by incubation at 60 °C into the SCPs.

Porcine collagen peptides were prepared from the porcine skin-derived gelatins by the following method. The porcine gelatins were dissolved by adding 20 times (*v*/*w*) the volume of distilled water and hydrolyzed by 0.2 wt % papain to the porcine gelatins at 50 °C for 5 h. After centrifugation at 4 °C, 10,000× *g* for 10 min, the supernatant was lyophilized. Then, lipids in the hydrolysates were removed by soaking in 99.5% ethanol for 3 h, and delipidated materials were dried by incubation at 60 °C into the porcine collagen peptides.

Egg white peptides were prepared from commercially available eggs by the following method. The egg whites were dissolved by adding twice the volume (*v*/*w*) of distilled water and hydrolyzed by 0.2 wt % papain to the egg whites collected from hens at 50 °C for 5 h. After centrifugation at 4 °C, 10,000× *g* for 10 min, the supernatant was lyophilized. Then, lipids in the hydrolysates were removed by soaking in 99.5% ethanol for 3 h, and delipidated materials were dried by incubation at 60 °C into the egg white peptides.

### 3.2. α-Glucosidase and Invertase Inhibitory Assays

Glucose quantitative chromogenic reagents were prepared by dissolving 5.3 mM 4-aminoantipyrine, 5.3 mM phenol, 2900 units (U) of glucose oxidase, and 355 U of peroxidase in 500 mL of 30 mM phosphate buffer solution (pH 7.4). The calibration curve was prepared by adding 20 μL of glucose solution (final concentration 0–500 mg/dL) to 3.0 mL of the chromogenic reagent, incubating at 37 °C for 30 min, and then measuring the absorbance at 505 nm. The curve was prepared for each lot of the chromogenic reagent.

The acetone powder (10 g), prepared from SD rat small intestine, was suspended in 0.1 M maleic acid buffer (pH 6.0) and sonicated. The supernatant was then centrifuged at 5 °C, 1007× *g* for 30 min. The supernatant obtained was used as crude α-glucosidase-invertase.

The α-glucosidase and invertase inhibitory activities were measured according to the method of Ohta et al. [37]. Seventy microliters of 0.1 M maleate buffer (pH 6.0), 10 µL of 500 mM maltose or sucrose solution, and 10 µL of the SCPs solution (final concentration at 0, 10, 50, 100, and 200 mg/mL) were mixed in vitro. To the mixed solution, 10 μL of the crude enzyme was added and incubated at 37 °C for 60 min to degrade maltose or sucrose, respectively. After incubation, the enzymatic reaction was stopped by adding 100 μL of 2.0 M maleic acid–Tris–NaOH buffer (pH 7.4), and the weight of glucose produced was determined by adding 3.0 mL of glucose quantification chromogenic reagent to 20 μL of the reacted solution. The inhibition rate for maltose and sucrose degradation was calculated using the following equation:

inhibitory activity (%) = [1 − (As − Asb)/(Ac − Acb)] × 100(1)

where As is the absorbance of the sample, Asb is the absorbance when the stop solution was added to the sample before the reaction, Ac is the absorbance of the buffer, and Acb is the absorbance when the stop solution was added to the buffer before the reaction.

### 3.3. DPP-IV Inhibitory Assay

DPP-IV inhibitory activity of the SCP solution (final concentration at 0, 1, 2, 3, 4, 5, and 6 mg/mL) was measured with the DPP-IV Drug Discovery Kit. One unit (U) of DPP-IV activity was defined as the amount of enzyme causing an increase of 2.09 × 10^−2^ in absorbance at 405 nm/min, based on the pre-measured results and using distilled water instead of the SCP solution. The IC_50_ was defined as an SCP concentration that inhibited 50% of 1 mU DPP-IV.

### 3.4. Measurement of Blood Glucose in OGTT

The OGTT was performed in accordance with the previous study [18]. Briefly, ICR mice or SD rats were divided into a control group and sample groups and fasted for 16 h. After basal blood collection (0 min), glucose solution (2 g/kg body weight, 15 mL/kg body weight) mixed with distilled water or with sample (1.5 g/kg body weight) were orally injected with a disposable probe (FUCHIGAMI, Kyoto, Japan). A single drop of blood was periodically sampled from the tail vein and glucose levels were measured using a glucose sensor (ACCU-CHEK ST meter, Roche DC Japan K.K., Tokyo, Japan).

### 3.5. Measurement of Glucose in the Stomach in OGTT

ICR mice were divided into a control group and an SCP group matched for body weight and fasted for 16 h. After being orally injected (0 min), the mice were sacrificed with CO_2_ gas and the stomachs were immediately extirpated with clamping distal ends of esophagus and pylorus. The stomachs were incised and flushed inside with 1 mL saline three times. Each stomach’s contents and the lavage fluids were mixed and centrifuged at 4 °C, 448× *g* for 10 min. The supernatants (20 μL) were mixed with 3.0 mL of the coloring reagent. After 30 min incubation at 37 °C, the absorbance at 505 nm was measured. Relative residual glucose content in the stomach:

(%) = S/C × 100(2)

where S is the glucose content in the stomach (g) and C is the dose of glucose (g).

### 3.6. Measurement of Glucose Content in Excrements in OGTT

ICR mice were divided into a control group and an SCP group matched for body weight and fasted for 16 h. Glucose solution mixed with sample or with distilled water was orally injected, and then the mice were fasted for 24 h. The mice could freely drink exchanged water throughout the test. The excrements that were excreted for 24 h after the oral administration were collected and lyophilized. After drying, the excrements were grinded and mixed with distilled water. Glucose in the mixtures was extracted by sonication at 20 °C, for 5 min. The extracts were centrifuged at 4 °C, 448× *g* for 10 min. The glucose concentration of the supernatants was measured as described in Section 2.4. Relative glucose content in the excrements:

(%) = S/C × 100(3)

where S is the glucose content in the excrements (g) and C is the dose of glucose (g).

### 3.7. Measurement of Blood Glucose and Insulin Levels in IPGTT

#### 3.7.1. Oral Administration of the SCPs

ICR mice were divided into a control group and an SCP group matched for body weight and fasted for 16 h. After basal blood collection (0 min), distilled water (control group) or SCP solution (0.3 g/mL, 1.5 g/kg body weight, 10 mL/kg body weight) (the SCP group) was orally administered with a disposable probe, and glucose solution (0.4 g/mL saline, 2.0 g/kg body weight, 5 mL/kg body weight) was injected intraperitoneally. A single drop of blood was periodically sampled (15, 30, 45, 60, and 120 min after the glucose injection).

#### 3.7.2. Intravenous Injection of the SCPs

ICR mice were divided into a control group and an SCP group matched for body weight and fasted for 16 h. After basal blood collection (0 min), saline (control group) or SCP solution (0.3 g/mL saline, 1.5 g/kg body weight, 5 mL/kg body weight) (the SCP group) was intravenously injected into the tail vein with a syringe, and glucose solution (0.4 g/mL saline, 2.0 g/kg body weight, 5 mL/kg body weight) was injected intraperitoneally. A single drop of blood was periodically sampled (15, 30, 60, and 120 min after the glucose injection).

### 3.8. Measurement of Insulin Levels

Blood samples (50 μL) were periodically drawn into a hematocrit tube coated with heparin (NRIS Micro hematocrit tubes Na-heparinized, 80 IU/mL), and poured into a tube containing aprotinin (final concentration at 500 KIU/mL) and heparin sodium (final concentration at 50 IU/mL). The blood samples were centrifuged at 4 °C, 2500× *g* for 10 min, and the plasmas were frozen at –80 °C until insulin measurement was taken. Plasma insulin levels were measured with LBIS Mouse Insulin ELISA Kit (AKRIN-011T, FUJIFILM Wako Shibayagi Corporation, Gunma, Japan).

In the SCP intravenous injection test, we collected whole blood from individual mice sacrificed periodically at each time point (0, 15, 30, 60, and 120 min after glucose injection) with a syringe containing heparin aprotinin and heparin sodium.

### 3.9. Measurement of Total GLP-1 Levels

Blood samples (125 μL) were drawn into a hematocrit tube coated with heparin at 0 and 15 min, and poured into a tube coated with EDTA 3K (final concentration at 1735 mg/mL) and heparin sodium (final concentration at 50 IU/mL). The blood samples were centrifuged at 4 °C, 2500× *g* for 10 min, and the plasmas were frozen at −80 °C until an insulin measurement was taken. Total GLP-1 levels in plasma were measured with a Multi Species GLP-1 Total ELISA kit (EZGLP1T-36K, Merck Millipore, Burlington, MA, USA).

### 3.10. Measurement of Endotoxin Concentration

The SCPs was dissolved in distilled water (endotoxin and β-glucan free) (Seikagaku Corporation, Tokyo, Japan) to a final concentration at 10 mg/mL. Endotoxin concentration was measured with an Endospecy^®^ ES-24S Kit. The potency of control standard endotoxin was 8 endotoxin units (EU)/ng from the certificate of analysis sheet.

### 3.11. Preparation of Type 1 Diabetes Model Rats

SD rats were divided into a control group and experimental groups matched for body weight. After basal collection under full feeding, saline (1 mL) (control group) or streptozotocin solution (1 mL) (70 mg/kg body weight, dissolved in 1 mL of saline) was intraperitoneally injected. After 4 days, blood glucose level under full feeding was measured, and rats with a level over 200 mg/dL were selected as type 1 diabetes model rats (STZ rats).

### 3.12. Statistical Analysis

The data in this study are expressed as mean ± standard error of the mean (SEM). Statistical analyses were performed as follows: using the Student’s *t*-test, the Mann–Whitney *U* test, and the Wilcoxon rank sum test. The significance test between the control group and the test group was the Student’s *t*-test. The significance test between the control group and multiple test groups, and between each test group, was the Tukey–Kramer test. All statistics were completed with *Statcel–The Useful Add-in Forms on Excel*, 3rd edition (OMS Publishing Inc., Tokorozawa, Japan).

## 4. Conclusions

We previously found that the SCPs prepared from the by-products of Bester sturgeon had an inhibitory effect on elevated blood glucose levels in the OGTT in mice. In this study, we investigated the mechanism of the SCP effect in detail. Consequently, (1) the SCPs did not inhibit α-glucosidase and invertase activities in an in vitro test, (2) the SCPs kept the glucose in the stomach longer in the OGTT, (3) the SCPs increased glucose excretion in the OGTT, (4) the orally administrated SCPs lowered the blood glucose level and stimulated the secretion of insulin in the IPGTT, (5) the intravenously injected SCPs lowered the blood glucose level and stimulated the secretion of insulin in the IPGTT, (6) the SCPs inhibited DPP-IV activity in an in vitro test, (7) the intravenously injected SCPs lowered the blood glucose level and maintained higher GLP-1 levels in the IPGTT, (8) the SCPs did not lower the blood glucose levels in rats without insulin secretory capacity (STZ rats). These results suggested that the following three mechanisms have an inhibitory effect on blood glucose caused by the SCPs: the SCPs slow the transportation rate of glucose from the stomach into the small intestine, resulting in delayed glucose absorption; the SCPs suppress the absorption of glucose in the small intestine and excrete it from the body; the SCPs inhibit DPP-IV in the blood and maintain a high GLP-1 level in the blood, which in turn stimulates insulin secretion.

## Figures and Tables

**Table 1:** Endotoxin concentration of SCPs prepared at different dates.

Lots	Endotoxin Concentrations
SCPs-1	0.014 ng/mg (0.112 endotoxin unit/mg)
SCPs-2	0.100 ng/mg (0.800 endotoxin unit/mg)

**Table 2:** Characteristics of DPP-VI inhibitory peptides.

Peptides	Peptides MW (Da)	Enzymes Used for Preparation	IC_50_ Values	
			(µg/mU)	(μmol/mU)
SCPs	1400 [18]	Papain	934	0.667
Collagen peptides (Atlantic salmon skin) [30]	<1000	Flavourzyme	270	0.270
Gly-Pro-Ala-Glu [30]	372.4	(Synthetic peptide)	0.924	0.248 × 10^−2^
Gly-Pro-Gly-Ala [30]	300.4	(Synthetic peptide)	1.26	0.210 × 10^−2^
Collagen peptides (tilapia scale) [22]	-	-	616	-
Collagen peptides (carp skin) [31]	2935	Esperase	425	0.145
	2322	Savinase	521	0.224
	1995	Alcalase	504	0.253
	3925	Trypsin	479	0.122
	5797	Izyme G	714	0.123
	2325	Protamex	467	0.201
	7878	Neutrase	529	0.067
	15,053	Peptidase	527	0.035
